# Dynamic Contrast-enhanced MR Imaging in Renal Cell Carcinoma: Reproducibility of Histogram Analysis on Pharmacokinetic Parameters

**DOI:** 10.1038/srep29146

**Published:** 2016-07-06

**Authors:** Hai-yi Wang, Zi-hua Su, Xiao Xu, Zhi-peng Sun, Fei-xue Duan, Yuan-yuan Song, Lu Li, Ying-wei Wang, Xin Ma, Ai-tao Guo, Lin Ma, Hui-yi Ye

**Affiliations:** 1 Department of Radiology, Chinese PLA General Hospital, Beijing, 100853, China; 2 Lift Science, Advanced Application Team, GE Healthcare China, Beijing, 100176, China; 3 Lift Science, Advanced Application Team, GE Healthcare China, Shanghai, 201203, China; 4 Department of Radiology, No.1 Hospital of Zhangjiakou, Hebei, 075000, China; 5 Medical Imaging Center, Jiayuguan Jiugang Hospital, Jiayuguan, Gansu, 735100, China; 6 Department of Radiology, General Hospital of Pingdingshan Coal Group, Pingdingshan, Henan, 467000, China; 7 Department of Urology, Chinese PLA General Hospital, Beijing, 100853, China; 8 Department of Pathology, Chinese PLA General Hospital, Beijing 100853, China

## Abstract

Pharmacokinetic parameters derived from dynamic contrast-enhanced magnetic resonance imaging (DCE-MRI) have been increasingly used to evaluate the permeability of tumor vessel. Histogram metrics are a recognized promising method of quantitative MR imaging that has been recently introduced in analysis of DCE-MRI pharmacokinetic parameters in oncology due to tumor heterogeneity. In this study, 21 patients with renal cell carcinoma (RCC) underwent paired DCE-MRI studies on a 3.0 T MR system. Extended Tofts model and population-based arterial input function were used to calculate kinetic parameters of RCC tumors. *Mean* value and histogram metrics (*Mode*, *Skewness* and *Kurtosis*) of each pharmacokinetic parameter were generated automatically using ImageJ software. Intra- and inter-observer reproducibility and scan–rescan reproducibility were evaluated using intra-class correlation coefficients (ICCs) and coefficient of variation (CoV). Our results demonstrated that the histogram method (*Mode*, *Skewness* and *Kurtosis*) was not superior to the conventional *Mean* value method in reproducibility evaluation on DCE-MRI pharmacokinetic parameters (*K*^ trans^ & *V*_e_) in renal cell carcinoma, especially for *Skewness* and *Kurtosis* which showed lower intra-, inter-observer and scan-rescan reproducibility than *Mean* value. Our findings suggest that additional studies are necessary before wide incorporation of histogram metrics in quantitative analysis of DCE-MRI pharmacokinetic parameters.

Dynamic contrast-enhanced magnetic resonance imaging (DCE-MRI), as a very common MRI technique, not only can subjectively judge the enhancement of a target area on a visual basis, semi-quantitatively characterize tumors using curvology^1^,^2^, but also can quantitatively evaluate parameters generated using pharmacokinetic models^3^,^4^ which reflected the dynamic distribution of Ga-related contrast agent in the different compartments of the tissue. The two-compartment model of DCE-MRI assumes the contrast agent exchanges between the plasma space and the extravascular-extracellular space (EES)^5^, and the forward and backward transfer rate could reflect the permeability of the microvasculature. It is used extensively in measuring tumor angiogenesis and blood brain barrier (BBB) disruption.

Pharmacokinetic DCE-MRI in oncology has been increasingly applied in quantitative scientific research and clinical practice. Zahra *et al*. recently summarized studies that have utilized DCE-MRI parameters to predict the efficacy of chemotherapy and concluded that DCE-MRI was a reasonably accurate and non-invasive method^6^.

Traditionally, many researchers utilize the mean value of the targeted region of interest (ROI) to perform analysis of tumors and made comparisons in the intra-observer, inter-observer, or test-retest analyses^7^,^8^,^9^,^10^. As a promising quantitative tool, the reliability and reproducibility of DCE-MRI suggests it will be widely used in future oncology analyses. Previously, we showed that the pharmacokinetic parameters of DCE-MRI in renal cell carcinoma (RCC) using *Mean* value of pharmacokinetic parameters demonstrated good reproducibility^11^.

However, beyond the tumor itself, much attention has been rightfully paid to tumor heterogeneity that exists in the tumor cell population due to the surrounding extracellular matrix, angiogenesis, and other tumor microenvironment features, all of which influence tumor characterization and therapeutic effect to a certain degree. Indeed, there is increasing interest in analyzing lesion heterogeneity by way of histogram analysis to characterize tumor subtypes^12^,^13^,^14^,^15^, tumor histological grades^16^,^17^,^18^,^19^, tumor aggressiveness^20^ and evaluate treatment effects^21^,^22^,^23^,^24^. This methodology has showed its utility in investigating the distributions of various tumor parameters such as permeability in dynamic contrast-enhanced MRI (DCE-MRI)^17^,^25^.

With the expected increase in use of heterogeneity analysis with DCE-MRI, it is therefore important to analyze its reproducibility capability before adopting its widespread use in performing analysis of tumor characterization or prediction of therapeutic effect. To the best of our knowledge, with the exception of a study by Heyes *et al*.^26^ that presented a histogram analysis approach combined with a semi-automatic lesion segmentation to show a decrease in inter-observer variability in the *K*^ trans^ parameter in DCE-MRI, no other studies have examined the reproducibility of histogram analysis. Herein, we evaluated the intra- and inter-observer, as well as scan–rescan reproducibility of histogram metrics in regard to DCE-MRI pharmacokinetic parameters in RCC.

## Methods

### Patients

Institutional Review Board of Chinese PLA General Hospital approved this prospective study. The methods used in this study were carried out in accordance with the Declaration of Helsinki. Written informed consent was obtained from each subject prior to study initiation. Patients with suspected renal cell carcinoma (RCC) during the imaging examinations were recruited from the urological clinic at our hospital from September 2012 to November 2012. Inclusion criteria were as follows: age ≥18 years old, glomerular filtration rate >60 mL/min, size of lesions >1.0 cm in diameter to avoid partial volume artifact concerns, and clear cell RCCs – as the most common pathologic subtype. Exclusion criteria included the following: common contraindication for MRI scans and the use of Ga-related contrast (such as metal implants, heart pacemaker, severe claustrophobia etc.), age <18 years old, glomerular filtration rate of <60 mL/min, size of lesions ≤1.0 cm in diameter, lesions with complete necrosis or cystic degeneration confirmed in MR examination, and patients with unacceptable DCE-MR imaging quality such as severe motion artifacts.

Sample size in this study was estimated using Power Analysis & Sample Size Software, PASS 11.0 (NCSS, LLC. Kaysville, Utah, USA). Due to usage of Intra-class Correlation Coefficient (ICC) as statistical tool and three observers in this study, we assumed the expected ICC of 0.9 (R1) and acceptable lowest ICC of 0.75 (R0), thus we set *α* = 0.05 and *β* = 0.20. Finally, through automatic calculation of PASS, the least acceptable number of subject (*k*) was 19.

### MRI technique

MRI scans were performed on a 3.0 T platform (GE Discovery MR 750, GE Healthcare, Milwaukee, WI) with an 8-channel surface phased-array coil. Patients were scanned twice with the first scan within 48 h of the initial diagnosis and the second scan at 48–72 h after the first scan, where the same lying position and scanning location were utilized. Breathing training was conducted before each scan. Besides routine scanning sequence (i.e., axial and coronal T2-weighted imaging), DCE-MRI was performed, which consisted of a pre-contrast T1 mapping sequence and a dynamic sequence. T1 mapping included multi-flip angles (3°, 6°, 9°, 12°, and 15°) pre-contrast scan with three-dimensional (3D) spoiled-gradient recalled-echo sequences for liver acquisition with volume acceleration (LAVA) in breath-hold mode. Dynamic sequence was performed with the same parameters as T1 mapping but with flip angle 12°, which resulted in a tempo resolution of 6 s. During dynamic scan, two successive phases for 12 s in a breath-holding mode and an interval for 6 s in a free-breathing mode were performed alternatively. The entire dynamic process lasted for 4.4 minutes. Scanning parameters were as follows: repetition time (TR) 2.8 *ms*, echo time (TE) 1.3 *ms*, matrix 288 × 180, field of view (FOV) 38 × 38 *cm*, slice thickness 6 mm, number of excitations (NEX) 1, bandwidth 125 kHz, and parallel imaging acceleration factor 3. When the scan for the third phase was started, the contrast media (0.1 mmol/kg, Omniscan, GE Healthcare) was administered intravenously as a bolus injection at a rate of 2 mL/s using a power injector (Spectris; MedRad, Warrendale, PA), followed with 20 mL normal saline flush at the same rate.

### Image post-processing and analysis

All images were transferred to an Omni-Kinetics workstation (GE Healthcare, LifeScience, China) for analysis. Non-rigid registration method suggested in literature^27^,^28^,^29^ was used to assess and correct medical image alignment within dynamic scans. The workstation used a framework (a free-form deformation algorithm) as previously described^30^,^31^,^32^ to help remove any error of misalignment between consecutive MRI scans, thus making our results more accurate than the non-processed images.

### Calculation of Pharmacokinetic Parameters

Multiple flip angles method^33^,^34^ was used to perform T1 mapping to obtain both the T1 value of the tissue before and after contrast agent injection using Equation 1, where *m*_0_ is the equilibrium signal intensity, *θ* is the flip angle, *TR* is the repetition time, T1 is the tissue T1 value, *S*(*θ*) is the T1 signal intensity. Then the contrast agent concentration in the tissue was computed using Equation 2^34^, where *T*_1_ is the T1 value after contrast injection, *T*_10_ is T1 value before contrast injection, and *r* (mM^−1^s^−1^) represents the longitudinal CA relaxation coefficient; thus, signal intensity of the tissue is converted to tissue CA concentration (*C*_t_(*t*)). The widely used two-compartment extended-Tofts model^35^ (Equation 3) with population averaged arterial input function (AIF)^33^,^34^ (Equation 4) was used to calculate the kinetic parameters. Where in Equation 3, *K*^ trans^ represented the transfer constant from plasma to the extracellular extravascular space (EES);  *V*_e_ represented the ratio of the EES volume to tissue volume; *V*_p_ represented the ratio of blood plasma volume to tissue volume;


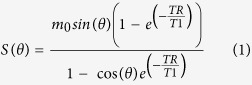



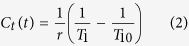



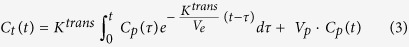



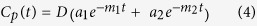


*K*_ep_ was the efflux rate constant from EES to plasma and equaled *K*^ trans^/*V*_e_; *C*_*t*_*(t)* and *C*_*p*_*(t)* represented the contrast agent concentrations in the tissue and blood plasma, respectively. In Equation 4, D = 1.0 mmol/kg, a_1_ = 2.4 kg/l, a_2_ = 0.62 kg/l, m_1_ = 3.0 and m_2_ = 0.016.

### ROI selection

**Figure 1:**
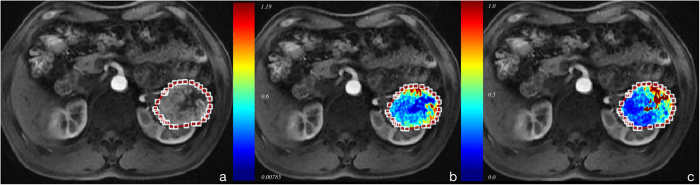
66-year-old male patient with 7.1 cm sized clear cell renal cell carcinoma in the left kidney. (**a**) Enhanced image on corticomedullary phase shows heterogeneous enhancement and necrosis. (**b**,**c**) Parametric maps of *K*^ trans^ and *V*_e_, respectively. The Mean value of *K* ^trans^ and *V*_e_ are 0.335 min^−1^ and 0.531, respectively.

Using reference information from anatomic axial and coronal T2-weighted images and post-contrast T1 images, the slice with the maximum diameter of the tumor was selected in the ImageJ software (National Institutes of Health, Bethesda, MD). Three radiologists (Z.S., F.D., Y.S., all board-certified radiologists engaged in abdominal imaging for 8, 10, 9 years, respectively) outlined ROIs around the edges of the tumors on the DCE-MRI map (Fig. 1a). Parameter outlines covered the whole tumor as much as possible and excluded pulsatile artifacts from blood vessels and susceptibility artifacts from adjacent bowels. Then the same ROI was copied to parametric maps (Fig. 1b,c).

**Figure 2:**
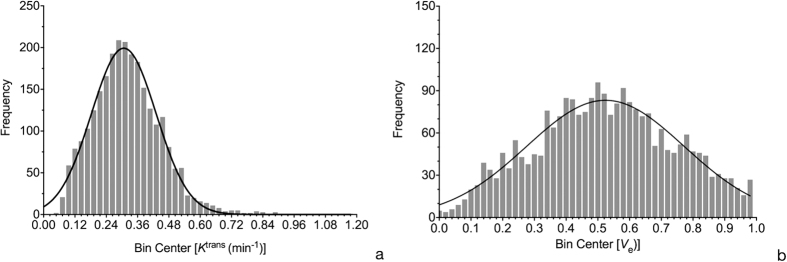
Histogram of pharmacokinetic parameters of clear cell RCC. (**a**) Histogram of *K *^trans^ shows that *Mean*, *Mode*, *Skewness* and *Kurtosis* are 0.335 min^−1^, 0.300 min^−1^, 1.100 and −0.2216, respectively. (**b**) Histogram of *V*_e_ shows that *Mean*, *Mode*, *Skewness* and *Kurtosis* are 0.531, 0.510, 0.0139, and −1.061, respectively.

Commonly, values of *K*^ trans^ greater than 1.2 min^−1^ are considered pseudo-permeability in large blood vessels or errors in fitting^36^,^37^; therefore any pixels with *K*^ trans^ larger than 1.2 min^−1^ or with *V*_e_ beyond the range of 0–100% were excluded from parametric maps. Based on this situation, histogram function in ImageJ was utilized and threshold value of kinetic parameters were set respectively such as *K*^ trans^ (0, 1.2 min^−1^), and *V*_e_ (0, 1). Then the traditional *Mean* values of *K*^ trans^, and *V*_e_ and heterogeneity analysis (i.e., *Mode, Skewness,* and *Kurtosis*) were automatically calculated. *Kurtosis* described how sharply peaked a histogram was compared with the histogram of a normal distribution. Accordingly, whereas a normal distribution had a *Kurtosis* of 0, a more peaked histogram had a positive *Kurtosis* value. *Skewness* described the degree of asymmetry of a histogram: a perfectly symmetric histogram had a *Skewness* of 0, a histogram with a long right tail had a positive *Skewness*, whereas a negative *Skewness* was due to the presence of a long left tail. The histogram graphs were plotted with the parametric values on the x-axis with a bin size of 0.024 min^−1^ for *K*^ trans^, and 0.02 for *V*_e_ (with a bin number of 50) (Fig. 2a,b).

The first observer (Z.S.) measured parameters for the first MRI examination twice (for intra-observer reproducibility) and observers 2 (F.D.) and 3 (Y.S.) measured the parameters of the first examination once (to examine inter-observer reproducibility). Then the first observer measured parameters of the second examination once (for scan–rescan reproducibility), carefully choosing the same slice as in the first scan or as close as possible.

### Statistical Analyses

#### Intra-, inter-observer, and scan–rescan differences in histogram metrics of kinetic parameters

Intra-observer and inter-scan differences were assessed using paired *t* tests. Inter-observer differences were evaluated using ANOVA.

#### Intra-, inter-observer, and scan–rescan agreement analyses in histogram metrics of kinetic parameters

Intra-observer, inter-observer, and scan–rescan agreements of histogram metrics of pharmacokinetic parameters were evaluated using the inter-class correlation coefficient (*ICC*). The agreement was defined as good (*ICC* > 0.75), moderate (*ICC* = 0.5–0.75), or poor (*ICC* < 0.5).

#### Intra-, inter-observer, and scan–rescan variability in histogram metrics of kinetic parameters

Coefficients of variation (CoV) were computed as the proportion of the standard deviation of the mean (standard deviation/mean, expressed as percentage). For CoVs describing the inter-observer variability, standard deviation was computed over each parameter obtained by all three observers. For CoVs concerning the intra-observer variability, standard deviation was computed over two measurements by each observer. For scan-rescan variability, the CoV for each subject was first computed and then averaged to obtain mean between patients’ CoVs for each parameter.

All statistical analyses were performed with SPSS software (IBM SPSS Statistics for Macintosh, Version 22.0. Armonk, NY: IBM Corp.) and GraphPad Prism (ver. 6.0; GraphPad Software, Inc., La Jolla, CA). *P* values <0 .05 were considered to indicate a statistically significant difference.

## Results

### Patients and lesions characteristics

A total of 28 patients with renal lesions underwent DCE-MRI scanning. After reviewing imaging quality and histopathologic results, two cases were excluded due to poor imaging quality and five cases due to other tumor types (1 papillary RCC, 3 chromophobic RCC, and a renal angiomyolipoma). Thus, 21 effective paired data sets of clear cell RCC cases (17 male, 4 female; age range 37–69 years, mean age 54.6 years; mean tumor size, 5.0 ± 2.2 cm) were included in this study.

### Histogram metrics of pharmacokinetic parameters of renal cell carcinoma

*Mean*, *Mode*, *Skewness*, *Kurtosis* of *K*^ trans^ and *V*_e_ of each ROI of 21 patients were automatically calculated and recorded. Then all *Mean*, *Mode*, *Skewness*, *Kurtosis* were documented for intra-observer, inter-observer and scan-rescan comparison in Table 1.

### Analysis of differences in kinetic parameters

There were no statistically significant intra-observer or inter-observer differences in any histogram metrics of each kinetic parameter examined, nor between MRI scan (all *P* > 0.05) (Table 1).

### Agreement analysis

#### Intra- and inter-observer agreement

The intra-observer ICCs of histogram parameters and *Mean* of kinetic parameters were all greater than 0.80, which indicated good-to-excellent agreements (range, 0.824–0.999; *P* < 0.001) (Table 2). The inter-observer ICCs of *Mean*, *Mode* and *Skewness* of *K*^ trans^ demonstrated excellent agreement while *Kurtosis* of *K *^trans^ showed moderate agreement (ICC, 0.728; 95%CI, 0.454~0.902). The inter-observer ICCs of histogram parameters and *Mean* of *V*_e_ showed good-to-excellent agreement (range, 0.828~0.968; *P* < 0.001). The ICCs details are listed in Table 2. Moreover, in both intra- and inter-observer agreement analyses, *Mode*, *Skewness,* and *Kurtosis* showed slightly lower ICCs than *Mean*.

#### Scan-rescan agreement

ICC of all histogram parameters of *V*_e_ showed good agreement (range, 0.758~0.798, *P* < 0.001) and showed similar ICCs with *Mean*. However, *Mean*, *Mode* of *K *^trans^ showed moderate agreement, *Skewness* and *Kurtosis* of *K*^trans^ showed poor agreement (0.352, 0.308, respectively). The ICCs in details was listed in Table 2.

### Variability analysis

#### Intra- and inter-observer variability

**Figure 3:**
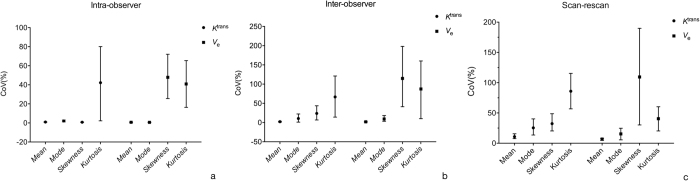
Variability analysis. (**a**)The intra-observer CV (%) values of *Mean*, *Mode*, *Skewness* and *Kurtosis* of *K *^trans^ and *V*_e_. (**b**) The inter-observer (%) values of *Mean*, *Mode*, *Skewness* and *Kurtosis* of *K*^ trans^ and *V*_e_. (**c**) The scan-rescan CV (%) values of *Mean*, *Mode*, *Skewness* and *Kurtosis* of *K*^ trans^ and *V*_e_. All data are presented as mean and 95% confidence interval.

In both intra- and inter-observer analysis, *Mean* of *K*^trans^ and *V*_e_ showed small variation (<=2.31%), *Mode* showed a larger variation (up to 10.54%), and *Skewness* and *Kurtosis* showed much higher CoVs than *Mean* (Fig. 3a,b) except for *Skewness* of *K*^trans^ in intra-observer analysis.

#### Scan-rescan variability

In scan-rescan analysis, *Mean* of *K *^trans^ and *V*_e_ showed small variation (10.82% and 6.88% respectively), *Mode* of *K *^trans^ and *V*_e_ showed relatively larger variation (25.44% and 15.43% respectively); however, *Mode*, *Skewness* and *Kurtosis* demonstrated larger variation, especially for *Skewness* and *Kurtosis* (>30%) (Fig. 3c).

**Figure 4:**
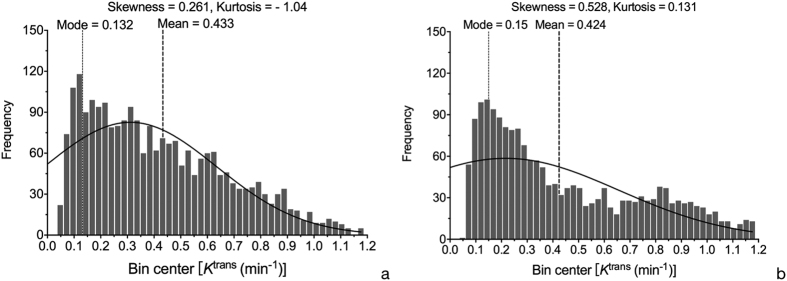
Histogram comparison of K^ trans^ between two DCE-MRI scans (Fig. 4a. 1st scan; Fig. 4b. 2nd scan). Although *Mean* value of *K *^trans^ of two scans is similar, *Skewness* and *Kurtosis* demonstrate obvious difference.

In addition, when comparing scan-rescan performance with intra- and inter-observer performance, the former variation was greater than the latter (Table 3) for nearly all histogram metrics of both *K *^trans^ and *V*_e_. In scan-rescan analysis, *Mean* value of pharmacokinetic parameters was similar between the two scans, and *Skewness* and *Kurtosis* showed obvious difference (Fig. 4a,b).

## Discussion

In this study, we found that scan-rescan performance had a relatively poorer reproducibility than intra- and inter-observer analysis regarding to histogram metrics of DCE-MRI pharmacokinetic parameters (*K *^trans^ & *V*_e_) in RCC. As for agreement analysis, scan-rescan ICCs of all histogram parameters were lower than intra- and inter-observer ICCs and intra-observer performance showed the highest ICCs. This suggested that although we attempted to ensure the situations were identical between the 1st and 2nd scan, it was unavoidable that minute differences in biological elements and/or hardware situation persisted between two scans, which likely resulted in more variation than difference of observers or drawing ROI.

In analyzing the variability results, scan-rescan variation for most of parameters was higher than intra- and inter-observer variation. However, *Skewness* and *Kurtosis* of *V*_e_ in inter-observer analysis showed the largest variation, which probably indicated that the observers exerted relatively great influence on measurement of these two values. In another aspect, when making comparison among the four histogram metrics of pharmacokinetic parameters regarding to reproducibility, we found that *Mean* and *Mode* presented better reproducibility than *Skewness* and *Kurtosis* in intra-, inter-observer and scan-rescan performance. These results showed that although heterogeneity analysis has been a trend in quantitative image analysis, it may not be as reproducible as standard *Mean* value analysis.

In examining intra- and inter-observer agreement, *Mean* of *K*^ trans^ and *V*_e_ demonstrated good agreement (all ICC values >0.75). Similar results were previously reported by Davenport *et al*.^38^ (i.e., inter-observer agreement: 0.88 and 0.87 ICCs for *K*^ trans^ and *V*_e_, respectively) and a study by Braunagel *et al*. also on RCC (ICC ranging from 0.79~0.97 *K *^trans^, *K*_ep_, and *V*_p_ in both intra- and inter-observer agreement)^39^. In scan-rescan agreement analysis, *Mean* of V_e_ showed good agreement (ICC, 0.764), which was in accordance with previous studies in gliomas^37^ and uterine fibroids^40^.

However, for *K*^trans^ alone, *Skewness* and *Kurtosis* demonstrated markedly lower ICCs and higher variation than *Mean* and *Mode* except for *Skewness* in intra-observer analysis. Additionally, for *V*_e_ alone, although ICC analysis showed similar result, variation of *Skewness* and *Kurtosis* were much higher than *Mean* and *Mode*. It is not clear why *Skewness* and *Kurtosis* were relatively poorly reproducible than *Mean* and *Mode*. We posit that the former was more sensitive to human interference (intra-observer), experience (inter-observer), and change of situation (scan-rescan) than the latter. However, we cannot rule out the likelihood that *Skewness* and *Kurtosis* were probably more sensitive to minute tumor changes.

Furthermore, we demonstrated that when comparing *K*^trans^ with *V*_e_, *Mean* of *V*_e_ had better reproducibility than *K*^trans^, which we also observed in our prior study study^11^. However for *Skewness* and *Kurtosis*, *V*_e_ and *K*^trans^ showed poor reproducibility except for *Skewness* in intra-observer analysis.

During parameter extraction, the most sensitive method to a dynamic scan’s temporal resolution is AIF. Personal or individual AIF if calculated accurately can improve performance of pharmacokinetic parameters, however, personal AIF requires a high temporal resolution and may be influenced by patients’ physiological condition, ROI placement, partial volume effect and inflow effect etc. So it is almost impossible to have an identical AIF when performing scans twice in the same patient. Due to non-continuous scanning mode of DCE-MRI (See “MRI technique” in Methods) for balancing the needs of clinical practice and scientific research, the temporal resolution of DCE-MRI was limited in this study. These facts led us to use a population-based AIF method, rather than a personal AIF. Population-based AIF not only helped address temporal resolution difficulties but also reduced AIF ROI location and sizing errors that have been reported previously^41^. In addition, the population-based AIF works equally well as the individual AIF for estimating pharmacokinetic parameters, as confirmed by several investigators^42^,^43^,^44^.

In our study we performed the DCE-MRI scan on a 3.0-Tesla MRI system. When compared with 1.5- or 1.0-Tesla, 3.0-Tesla DCE-MRI presented higher SNR and faster scan speed (potentially increasing temporal resolution) which therefore benefit DCE-MRI performance. However, 3.0-Tesla DCE-MRI increased potency of magnetic susceptibility and chemical shift, especially susceptibility to air artifacts. Hence, it is not recommended that 3.0-Tesla DCE-MRI was used to evaluate tumors adjacent to air or gas^45^.

This study has a few limitations. Firstly, we analyzed only single slices of tumor. Although it is reported that the efficacy was similar with whole tumor analysis, this method will likely exclude some information reflecting on the whole tumor characteristics. However, whole tumor analysis is very time-consuming and manual ROI allocation on all slices may increase measurement error. Secondly, besides the histogram parameters we used, histogram metrics covers many more aspects. In this study, we only analyzed a portion of histogram metrics, *Median, Percentiles,* and *Texture* parameters (uniformity and entropy) were not taken into consideration; but we included the descriptive parameters and distribution parameters such as *Skewness* and *Kurtosis*, which can adequately analyze the average value and heterogeneity to a certain degree. Thirdly, we used renal tumor as an example to compare histogram metrics to conventional *Mean* value analysis. Potentially, these results cannot be generally extended to other types of tumors derived from other anatomical sites. Further studies and exploration of other tumors are therefore required.

In conclusion, histogram method (*Mode*, *Skewness* and *Kurtosis*) was inferior to the conventional *Mean* value method in reproducibility evaluation on DCE-MRI pharmacokinetic parameters (*K*^trans^ & *V*_e_) in renal cell carcinoma, which suggests that histogram analysis may not be appropriate for quantitative evaluation of DCE-MRI pharmacokinetic parameters in renal cell carcinoma at present.

## Additional Information

**How to cite this article**: Wang, H.-y. *et al*. Dynamic Contrast-enhanced MR Imaging in Renal Cell Carcinoma: Reproducibility of Histogram Analysis on Pharmacokinetic Parameters. *Sci. Rep.* **6**, 29146; doi: 10.1038/srep29146 (2016).

## Figures and Tables

**Table 1:** Histogram metrics of pharmacokinetic parameters of DCE-MRI and analysis on difference.

Kinetic Parameters	Histogram Metrics	Intra-observer			Inter-observer				Scan-rescan		
		1^st^ Measurement	2^nd^ Measurement	*P* value	Observer 1	Observer 2	Observer 3	*P* value	1^st^ Scan	2^nd^ Scan	*P v*alue
*K*^trans^	*Mean* (min^−1^)	0.466 ± 0.140	0.465 ± 0.145	0.878	0.466 ± 0.140	0.457 ± 0.132	0.461 ± 0.137	0.986	0.466 ± 0.140	0.450 ± 0.092	0.581
	*Mode* (min^−1^)	0.370 ± 0.194	0.372 ± 0.189	0.754	0.370 ± 0.194	0.374 ± 0.196	0.402 ± 0.170	0.899	0.370 ± 0.194	0.325 ± 0.128	0.306
	*Skewness*	0.622 ± 0.396	0.613 ± 0.374	0.638	0.622 ± 0.396	0.575 ± 0.281	0.654 ± 0.336	0.850	0.622 ± 0.396	0.653 ± 0.389	0.870
	*Kurtosis*	−0.752 ± 0.494	−0.758 ± 0.512	0.927	−0.752 ± 0.494	−0.532 ± 0.658	−0.878 ± 0.362	0.268	−0.752 ± 0.494	−0.708 ± 0.543	0.854
*K*_ep_	*Mean* (min^−1^)	0.822 ± 0.353	0.835 ± 0.352	0.339	0.823 ± 0.353	0.833 ± 0.358	0.839 ± 0.368	0.972	0.823 ± 0.353	0.760 ± 0.347	0.160
	*Mode* (min^−1^)	0.600 ± 0.300	0.550 ± 0.266	0.339	0.600 ± 0.300	0.525 ± 0.186	0.599 ± 0.300	0.732	0.600 ± 0.300	0.550 ± 0.266	438
	*Skewness*	4.634 ± 1.398	4.671 ± 1.370	0.083	4.634 ± 1.398	4.490 ± 1.720	4.532 ± 1.215	0.971	4.634 ± 1.398	5.121 ± 1.206	0.097
	*Kurtosis*	23.593 ± 13.392	23.954 ± 13.166	0.111	23.593 ± 13.392	23.126 ± 14.327	22.271 ± 12.777	0.971	23.593 ± 13.392	28.691 ± 12.979	0.104
*V*e	*Mean* (min^−1^)	0.559 ± 0.107	0.558 ± 0.105	0.651	0.559 ± 0.107	0.551 ± 0.116	0.553 ± 0.118	0.985	0.559 ± 0.107	0.576 ± 0.107	.0423
	*Mode* (min^−1^)	0.508 ± 0.231	0.511 ± 0.230	0.491	0.508 ± 0.231	0.517 ± 0.229	0.517 ± 0.216	0.995	0.508 ± 0.231	0.578 ± 0.224	0.116
	*Skewness*	0.330 ± 0.370	0.386 ± 0.476	0.253	0.330 ± 0.370	0.290 ± 0.467	0.290 ± 0.425	0.730	0.330 ± 0.370	0.231 ± 0.572	0.322
	*Kurtosis*	−0.692 ± 0.485	−0.577 ± 0.619	0.245	−0.692 ± 0.485	−0.712 ± 0.581	−0.623 ± 0.640	0.928	−0.692 ± 0.485	−0.722 ± 0.746	0.816

**Table 2:** ICC analysis on histogram metrics of pharmacokinetic parameters of DCE-MRI.

Kinetic Parameters	Histogram Metrics	Intra-observer		Inter-observer		Scan-rescan	
		*ICC*(95%CI)	*P* value	*ICC*(95%CI)	*P* value	*ICC*(95%CI)	*P* value
*K*^ trans^	*Mean*	0.999 (0.996, 1.000)	<0.001	0.993 (0.981, 0.998)	<0.001	0.686 (0.212, 0.898)	0.006
	*Mode*	0.994 (0.980, 0.998)	<0.001	0.923 (0.816, 0.975)	<0.001	0.616 (0.121, 0.870)	0.001
	*Skewness*	0.985 (0.951, 0.996)	<0.001	0.898 (0.761, 0.966)	<0.001	0.352 (−0.288, 0.762)	0.863
	*Kurtosis*	0.929 (0.770, 0.979)	<0.001	0.728 (0.454, 0.902)	<0.001	0.308 (−0.346, 0.743)	0.767
*V*_e_	*Mean*	0.998 (0.993, 0.999)	<0.001	0.991 (0.976, 0.997)	<0.001	0.764 (0.378, 0.925)	0.001
	*Mode*	0.999 (0.998, 1.000)	<0.001	0.934 (0.837, 0.979)	<0.001	0.758 (0.370, 0.923)	0.001
	*Skewness*	0.925 (0.769, 0.977)	<0.001	0.945 (0.950, 0.994)	<0.001	0.766 (0.390, 0.926)	0.001
	*Kurtosis*	0.824 (0.517, 0.945)	<0.001	0.895 (0.755, 0.965)	<0.001	0.780 (0.562, 0.932)	0.001

**Table 3:** Variability analysis on histogram metrics of pharmacokinetic parameters of DCE-MRI.

Kinetic Parameters	Histogram Metrics	Coefficient of variation (%)		
		Intra-observer	Inter-observer	Scan-rescan
*K*^ trans^	*Mean*	0.98	2.31	10.82
	*Mode*	2.10	10.54	25.44
	*Skewness*	0.73	23.84	32.29
	*Kurtosis*	42.22	66.72	85.84
*V*_e_	*Mean*	0.72	1.84	6.88
	*Mode*	0.66	9.21	15.43
	*Skewness*	47.87	114.86	109.42
	*Kurtosis*	40.92	87.36	40.53
